# Effect of checklist based box system interventions on improving institutional delivery among reproductive age women in Northwest Ethiopia: generalized structural equation modeling

**DOI:** 10.1186/s13690-021-00774-2

**Published:** 2022-01-04

**Authors:** Netsanet Belete Andargie, Gurmesa Tura Debelew

**Affiliations:** 1 Ministry of Health, Addis Ababa Ethiopia and Department of Population and Family Health, Jimma University, Jimma, Ethiopia; 2 Department of Population and Family Health, Jimma University, Jimma, Ethiopia

**Keywords:** Box system, Institutional delivery, Generalized structural equation modeling, Northwest Ethiopia

## Abstract

**Background:**

Previous studies have shown that there is low utilization of institutional delivery in Ethiopia, as well as various factors contributing to this low utilization. Notably, there is paucity around interventions to improve institutional delivery. Hence, this study examines the effectiveness of checklist-based box system intervention on improving institutional delivery and to investigate the association through which the intervention is linked to institutional delivery.

**Method:**

The study used data from a larger trial, on the effectiveness of checklist-based box system intervention on improving maternal health service utilization. In the intervention arm, mothers received regular community-level pregnancy screening and referral, service utilization monitoring boxes, drop-out tracing mechanisms, regular communication between health centers and health posts, and person-centered health education for mothers. This study used the existing government-led maternal health program as a control arm. A total of 1062 mothers who gave birth one-year before the survey were included in the final analysis. A difference-in-difference estimator was used to test the effectiveness of the intervention. Generalized structural equation modeling was used to examine the direct and/ indirect associations between the intervention and institutional delivery.

**Result:**

Among participants, 403 (79.5%) mothers from intervention and 323 (58.2%) mothers from control clusters gave birth at health facilities. The result of the study revealed a 19% increase in institutional delivery in the intervention arm (19, 95%CI: 11.4-27.3%). In this study the pathway from checklist-based box system intervention to institutional delivery was mainly direct - (AOR = 3.32, 95%CI: 2.36-4.66), however, 33% of the effect was partially mediated by attendance of antenatal care four visits (AOR = 1.39, 95%CI: 1.02-1.92). The influence of significant others (AOR = 0.25, 95%CI: 0.15-0.43) and age (AOR = 0.03, 95%CI: 0.01- 0.09) had an inverse relation with institutional delivery.

**Conclusion:**

The implementation of a checklist-based box system significantly increased institutional delivery utilization, both directly and indirectly by improving antenatal care four attendance. A larger-scale implementation of the intervention was recommended, taking the continuum of care approach into account.

**Trial registration:**

ClinicalTrials.gov, NCT03891030, Retrospectively registered on 26 March, 2019.

**Supplementary Information:**

The online version contains supplementary material available at 10.1186/s13690-021-00774-2.

## Background

The World Health Organization (WHO) recommends that all pregnant women receive quality care throughout their pregnancy, childbirth, and postnatal period. WHO prioritized emergency obstetric care in its safe motherhood initiative, with the goal of ensuring safe birth and reducing actual and potential complications for the mother and newborn [1–4]. Studies showed that institutional delivery could reduce the occurrence of maternal death by 16-33% [5, 6]. However, in developing countries most deliveries occur at home without skilled care assistance [7, 8]. Evidence from the 2019 Ethiopian Health and Demographic Survey (EDHS) showed that only 48% of women went to health facilities for childbirth services [9].

Institutional delivery was influenced by previous maternal health care visits that a mother had [10]. Studies also recommend that, unless antenatal care (ANC) becomes a bridge for having institutional deliveries, it may not fully achieve its objective as major life-threatening obstetric complications commonly occur during and after delivery [11, 12]. Previous studies in Ethiopia investigate the magnitude of institutional delivery, and most reported it as ‘low’ [13–16], compared to the country level goal for 2020, which was 90% [17]. Works of literatures also identified several modifiable factors associated with institutional delivery such as attitude towards institutional delivery, maternal age, frequency of antenatal care visits, antenatal depressive symptoms, attending pregnant mother conferences etc. However, in Ethiopia, there are limited interventional studies conducted to improve utilization of institutional delivery. A pragmatic study on the effectiveness of Maternity waiting homes (MWHs), which are home-like environments located near health centers for near-term pregnant mothers, combined with community mobilization by trained local leaders in improving institutional births, revealed a small but non-significant improvement in institutional delivery [18]. Another intervention on the effect of m-health intervention on institutional delivery and postnatal care utilization involves, reminders of subsequent visits of ANC, delivery and PNC as well as educational messages on dangers signs during pregnancy and common complaints during pregnancy. This message in the pregnancy period was discovered to improve utilization of institutional delivery and postnatal care [19]. Another community trial to improve institutional delivery is the deployment of trained community-based nurses to rural communities in Ethiopia to assess SBA uptake levels. This study found that sending trained reproductive health nurses to rural Ethiopian communities increased SBA service utilization significantly [20].

Hence, checklist based box system intervention (CBBSI) was designed with the aim of contributing to improved maternal health care utilization, including institutional delivery. The intervention focused on the continuum of maternal health care and assessed the impact of previous maternal health visits (attendance of antenatal care four visit) on institutional delivery. This can also be implemented in both rural and urban settings, regardless of mobile phone ownership. The intervention was implemented with a two-pronged approach of demand creation and service utilization monitoring through drop-out tracing mechanisms. The intervention provided community-level pregnancy screening to identify suspected pregnant women and link them to a nearby health center. After the pregnancy was confirmed at the health center, the mother received her first ANC and became a part of the “service utilization monitoring box,” which was designed to track service utilization from the first antenatal care visit to the third postnatal care visit. This service utilization monitoring box assists midwives in identifying mothers who fail to attend recommended maternal health care visits. Through health education, health extension workers, in which the dropping mother belong, were able to sway her to return to service. The “health education scheduling box” aided health extension workers in prioritizing health education topics during this process [21].

In this study, the effectiveness of checklist based box system interventions on improving utilization of institutional delivery was assessed. In addition, the contribution of previous maternal health care visits including frequency of antenatal care visit to institutional delivery have been reported by many studies [14, 15, 22–24]. Therefore, this study also assessed the indirect paths through which the intervention is linked to institutional delivery.

## Methods

This trial was retrospectively registered on ClinicalTrials.gov with trial identifier NCT03891030 on March 26, 2019, and the trial protocol was published [21].

### Study design and setting

A double-blind, parallel-group, two-arm cluster randomized controlled trial was conducted to assess the effectiveness of checklist-based box system interventions on improving utilization of institutional delivery. The study was conducted in three districts of East Gojjam Zone: Debremarkos, Gozamin, and Machakel. East Gojjam zone is one of the administrative Zones of the Amhara region located in Northwest Ethiopia. According to the 2007 census, in the Amhara region, the total population was 2,153,937 and of these 1,087,221 were female [25]. In the region, 82.6, 55.7, 39.8% of mothers received at least one antenatal care, institutional delivery, and postnatal care, respectively [9]. Currently in Ethiopia, focused antenatal care (four visit model) is being implemented at the national level. This study was conducted from January 2019-September 2020.

### Study population and inclusion criteria

The study considers mothers of reproductive age group (15-49 years) as a source population. Similarly, mothers who gave birth last 1 year preceding the survey and part of checklist-based box system intervention trial were included.

### Sample size determination and sampling procedure

To get a more stable parameter estimates, the sample size for this study was calculated based on the estimable parameter to respondent ratio of 1:30 [26]. Accordingly, the number of estimable parameters were 26, which made the total sample size required to be 780. However, as this study is part of a larger trial, data from 1062 participants were included in the final analysis.

Regarding the sampling procedure, of the 16 districts available in East Gojjam Zone: Debre-Markos, Gozamin, and Machakel were selected on the basis of confirming that health posts/health centers within these districts didn’t receive an intervention/project aiming to improve utilization of maternal health services. Then a random sampling method was used to select health posts that were included in the study. The list of mothers who gave birth within 1 year preceding the survey was accessed from the health post’s family folder, where the profile of the residents in the catchment area of the health post was registered in. Then this list was entered into SPSS and taken as a sampling frames for the study. Then the required numbers of mothers were selected from the list using SPSS random selection command.

### Intervention

Checklist-based box system intervention is a service utilization improvement project aiming to improve the utilization of maternal health services: antenatal care, institutional delivery, and postnatal care. The intervention consisted of both demand creation and service utilization monitoring components. Mothers below 16 weeks of gestation were enrolled in the study and followed until they reached their postnatal care three visits. In this process, mothers who belong to the intervention group received: community-level pregnancy screening, then suspected pregnant mothers were linked with the nearby health center for laboratory confirmation of the pregnancy. Mothers with confirmed pregnancy received the first ANC on the same day, then service utilizations were monitored for each and every mother using a service utilization monitoring box placed at health centers. Mothers who drop the service before getting the third postnatal care had a reminder visit by health extension workers to resume their visits. The second box was placed at health posts where demand creation activities were scheduled. Health education topics were determined based on the gap that health extension workers identified during their community survey. The detailed procedure of the intervention process can be accessed from the published study protocol [21].

### Variables and measurement

- Institutional Delivery – Participants were asked ‘*where their last childbirth took place?’* Response were ‘0 for Home’ or ‘1 for Health Facility’.
- Month of ANC initiation – participants were asked about the time they initiated their ANC visits/receive the first antenatal care, and responses were less than or equal to 16 weeks and greater than 16 weeks of gestation.
- Antenatal care four visits– Participants were asked *‘How many antenatal care visits did they attend for their index pregnancy?’* This was recoded as ‘four or more visits’ and ‘less than four visits’. Institutional delivery, month of ANC initiation and antenatal care four visits were treated as endogenous observed variables in the generalized structural equation model.
- Checklist Based Box system Intervention – This was examined as whether the participant belongs to an intervention or a control wing.
- Social Support – Social support was measured using the following 14 item questions with ‘yes’ or ‘no’ response categories: ‘gets visits from significant others’, ‘getting useful advises’, ‘discussion on problems’, ‘having care at the time of labor and delivery’, ‘feeling loved’, ‘others thankful on them’, ‘getting help on household chores’, ‘help with money at emergency’, ‘help in transportation’, ‘help when sick’, ‘attending community level discussions’, ‘member of any religious cast’, ‘attending public meetings’ and ‘help in case of conflicts’.
- Influence – Participants were asked whether they had a negative influences from significant others (mother, grandmother, mother, neighbors, and husband) in the process of using maternal health services. The responses were ‘yes’ or ‘no’. Checklist based box system intervention, social support and influence were treated as exogenous observed variables in the generalized structural equation model.
- Knowledge of danger signs of labor and delivery – this was measured using an item consisting of seven questions with ‘yes’ or ‘no’ response categories: ‘severe headache’, ‘vaginal bleeding’, ‘convulsion’, ‘high fever’, ‘loss of consciousness’, ‘retained placenta’ and ‘prolonged labor’.
- Birth preparedness and complication readiness - was examined as a latent construct consisting of five items with ‘yes’ or ‘no’ response category: ‘arranged emergency transport’, ‘arranged emergency funds’, ‘personal saving and how to access them’, ‘choose place of delivery/skilled attendant’ and ‘knows who the blood donor is’. Birth preparedness and complication readiness and knowledge of danger signs of pregnancy and delivery were treated as endogenous latent variables.
- Family Support **-** was examined as a latent construct consisting of five item questions with ‘yes’ or ‘no’ response categories: ‘Provided advice and support to deliver at health facilities’, ‘help to get emergency transport during labor’, ‘accompany during labor’, ‘help to care baby’, and ‘help to attend post-natal visits’. Family support was treated as exogenous latent variable.

### Hypothesized theoretical model

**Fig. 1:**
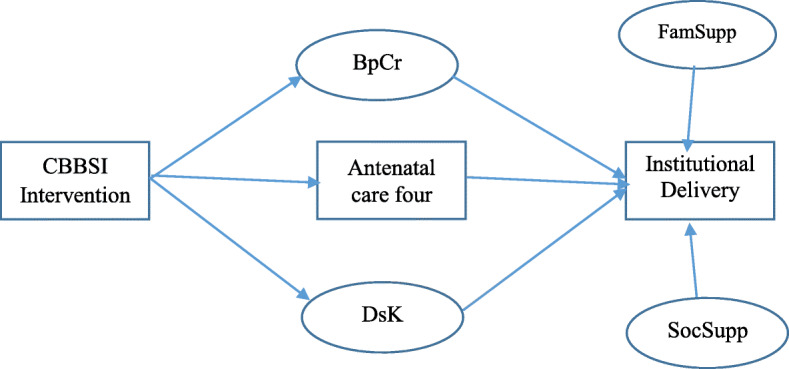
Hypothesized model for factors associated with institutional delivery: {Adjusted for age, month of initiation for the first ANC, influence}, January 2019-September 2020, Northwest Ethiopia (CBBSI-Checklist based box system intervention, BpCr-Birth Preparedness and complication readiness, DsK-Danger Signs Knowledge, FamSupp-Family support)

The hypothesized relationship between observed and latent variables with the outcome were constructed based on the findings from the literature review, the practicability of relationships, and authors’ experience. Then, potential pathways through which the intervention is linked to institutional delivery were created (Fig. 1).

**Fig. 2:**
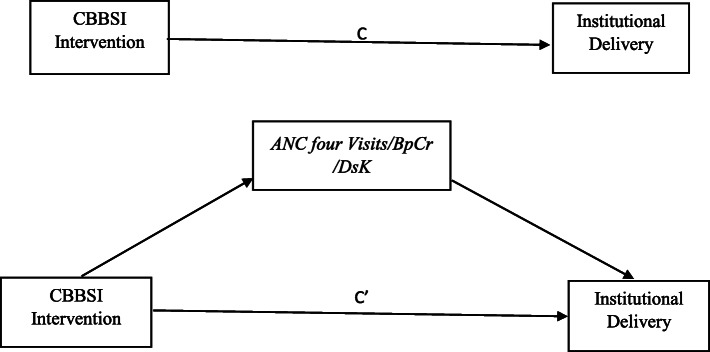
The effect of CBBSI on institutional delivery and the mediation effect of Antenatal four visits, birth preparedness and complication readiness and danger signs of labor and delivery, January 2019-September 2020, Northwest Ethiopia. (CBBSI-Checklist based box system intervention, BpCr-Birth Preparedness and complication readiness, DsK-Danger Signs Knowledge)

Previous studies reported that mothers attending four ANC visits were high likely to deliver at health facilities [14, 15, 22–24, 27, 28]. In addition to the direct relationship between the intervention and institutional delivery, pathways were created through potential mediators to institutional delivery (Fig. 2). In addition, studies conducted in South West and South East Ethiopia [8, 29] identified knowledge on danger signs of labor and delivery as one of the determinant factors for institutional delivery, similarly, mothers having birth preparedness and complication readiness practice more likely went for facility delivery as compared to their counterparts [24].

### Data processing, model building and analysis

Data was exported from Kobo Toolbox and imported to STATA Version 15 for analysis. Before the analysis, data were checked for missing values and outliers. Descriptive analysis was conducted to understand sample characteristics. Then, statically significant variables were identified by using bivariate analysis. The effectiveness of checklist-based box system intervention in improving institutional delivery was tested by using the difference in difference estimator.

Factor analysis was used to reduce the number of items per latent construct and to determine the contribution of each observed variable to the latent construct. Then, confirmatory factor analysis was used to see if the data fit the hypothesized measurement model and if there was a relationship between the observed variables and their underlying latent constructs. Accordingly, birth preparedness and complication readiness practice, knowledge of danger signs of labor and delivery and family support variables were treated as a latent variable in this analysis. For these latent variables, factor analysis was computed and factor reduction was done based on the factor loadings, taking 0.4 as a cutoff point. Then the relationship between each of the constructs with the latent variable was measured using generalized response confirmatory factor analysis. Similarly, factor analysis was also computed for social support, which was measured using a 14 item questions, a similar cut off point was applied.

Since this study contained a dependent variable ‘institutional delivery’ and various independent variables with generalized responses, Generalized Structural Equation Modeling (GSEM) was employed to examine the relationship between various exogenous and endogenous or mediating variables indicated in the hypothesized model (Fig. 1). This model was selected over multiple logistic regression models, because, logistic regression yields the direct effect of predictors on the outcome, but there might be variables that have a mediation effect (mediators) on the outcome.

Accordingly, institutional delivery, CBBSI, antenatal care four visit, month of ANC initiation, place of residence and influence of significant others had binary response categories and analyzed with Bernoulli family and logit link function. Social support was measured using 14 item questions. Initially social support was considered as a latent variable in the GSEM model. However, this increased model complexity and took longer time to converge. So, to get a model that best fits with the data, we compared two models: a model with social support as a latent variable and social support as a continuous variable. Social support was considered as a continuous variable through computing a raw sum scores, which produce a valid value ranging from 2 to 14. As the number of parameters to be estimated were reduced, considering social support as a continuous predictor produces a relatively less complex model. Then, social support was analyzed with Gaussian family and identity link function. Birth preparedness and complication readiness practice, knowledge of labor and delivery danger signs and family support were latent variables which constitute items with binary response categories, their measurement model was analyzed with Bernoulli family and logit link function. Age was treated as a continuous variable in the GSEM. Age and social support were categorized for the purpose of descriptive report.

The analysis was started with fitting the hypothesized model, and modifications were performed through adding theoretically supported path links. Finally a model with the minimum Akaike information criteria was retained. After the parameters were estimated, an indirect and total effects were computed through a non-linear combination of parameters [30]. The mediation effect was reported as complete (insignificant direct pathway), partial (significant indirect path) and no mediation (insignificant indirect pathways).

## Result

### Socio-demographic characteristics

**Fig. 3:**
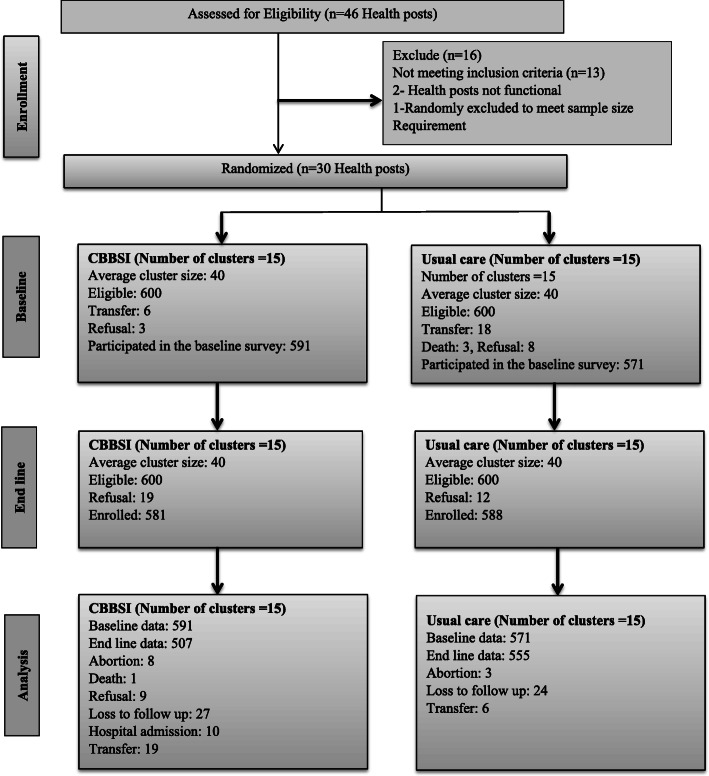
Participant flow diagram

Data from 1062 mothers who gave birth 1 year before the survey were included in the analysis (Fig. 3). The majority of participants were from rural residence 886 (83.4%), attended primary education 623 (58.7%) and were in marital union 1020 (96.1%) (Table 1).

**Table 1:** Description of Socio-demographic characteristics, January 2019-September 2020, Northwest Ethiopia

Variable	Intervention (*n* = 507)		Control (*n* = 555)		X^2^	*p-value*
	Freq.	%	Freq.	%		
Age					11.16	0.004
15-19	10	1.9	29	5.2		
20-29	352	69.4	345	62.2		
30-49	145	28.6	181	32.6		
Place of Residence					60.24	< 0.0001
Rural	376	74.2	510	91.9		
Urban	131	25.8	45	8.1		
Educational Status					32.34	< 0.0001
No formal Education	115	22.7	169	30.5		
Primary (1-8th grade)	293	57.8	330	59.5		
Secondary (9-12th grade)	62	12.2	49	8.8		
Above 12th Grade	377	74.4	7	1.3		
Marital Status					1.06	0.79
Single	6	1.2	10	1.8		
Married	490	96.6	530	95.5		
Separated	10	1.9	14	2.5		
Widowed	1	0.2	1	0.2		
Wealth Index					18.30	0.001
Poorest	199	39.3	207	37.3		
Poor	144	28.4	173	31.2		
Medium	73	14.4	116	20.9		
Rich	64	12.6	46	8.3		
Richest	27	5.3	13	2.3		
Parity					2.10	0.34
One Child	221	45.6	218	39.3		
2-4 Children	237	46.7	282	50.8		
≥ 5 Children	49	9.7	55	9.9		

### Effectiveness of checklist based box system intervention on improving institutional delivery

Of the participants, 432 (85.2%) mothers from intervention and 287 (51.7%) from control achieved antenatal care four visits for their index pregnancy. Similarly, 403 (79.5%) mothers from intervention and 323 (58.2%) from control gave birth at health facilities (Table 2).

**Table 2:** Description of maternal health service utilization, January 2019-September 2020, Northwest Ethiopia (Intervention (*n*) = 507, Control (*n*) = 555)

Variable	Intervention		Control		X^2^	*p-value*
	Freq.	%	Freq.	%		
Institutional Delivery					55.5	< 0.0001
Yes	403	79.5	323	58.2		
No	104	20.5	232	41.8		
Antenatal Care four Visit					135.9	< 0.0001
Yes	432	85.2	287	51.7		
No	75	14.8	268	48.3		
Month of the first ANC Initiation					0.98	0.32
≤ 16 weeks of gestation	193	35.1	162	32.1		
> 16 weeks of gestation	359	65.0	343	67.9		
Level of Birth Preparedness						
Arranged emergency transport (yes)	290	57.2	342	61.6	2.15	0.14
Personal saving and how to access them (yes)	258	50.9	297	53.5	0.73	0.39
Arranged emergency funds (yes)	272	53.6	279	50.3	1.21	0.27
Identify place of delivery/skilled attendant (yes)	214	42.2	181	32.6	10.44	0.001
Knows who the blood donor is (yes)	236	46.5	265	47.7	0.15	0.69
Knowledge of Danger signs of labor and Delivery						
Severe headache (yes)	473	93.2	522	94.1	0.26	0.61
Vaginal bleeding (yes)	356	70.2	299	53.9	29.94	< 0.0001
Convulsion (yes)	240	47.3	271	48.8	0.24	0.63
High Fever (yes)	235	46.4	281	50.6	1.94	0.16
Loss of Consciousness (yes)	244	48.1	245	44.1	1.69	0.19
Retained Placenta (yes)	224	44.2	212	38.2	3.91	0.04
Prolonged labor (yes)	254	50.1	299	53.9	1.51	0.23
Family Support						
Provided advise/support to deliver at health facilities (yes)	356	70.2	321	57.8	1.17	0.28
Help to get emergency transport during labor (yes)	321	63.3	368	66.3	3.64	0.05
Accompany during labor (yes)	292	57.6	328	59.1	1.47	0.22
Help to care my baby (yes)	402	79.3	358	64.5	24.91	< 0.0001
Help to attend post-natal visits (yes)	261	51.5	294	52.9	1.22	0.27
Level of Social Support					3.10	0.07
Good	344	67.9	404	72.8		
Poor	163	32.1	151	27.2		
Influence of Significant others					9.22	0.002
Yes	60	11.8	36	6.5		
No	447	88.2	519	93.5		

The difference-in-difference estimator showed that checklist-based box system intervention significantly improved institutional delivery in the study area (19, 95%CI: 11.4-27.3%). However, the lower confidence limit is slightly below the minimum difference that was planned to be detected between the intervention and control clusters, which was (12%).

### Factors associated with institutional delivery

The factor loadings for the whole measurement model turned significant at *p* value< 0.001 (Table 3), which showed the significance of items in measuring their respective latent constructs.

**Table 3:** Confirmatory Factor Analysis: unstandardized factor loadings, January 2019-September 2020, Northwest Ethiopia (*N* = 1062)

Constructs and indicators	Unstandardized factor loadings	*p-value*
Level of Birth Preparedness		*Cronbatch alpha = 0.88*
BpCr1 - Arranged emergency transport	1	
BpCr2 - Personal saving and how to access them	1.15	*< 0.0001*
BpCr3 - Arranged emergency funds	2.28	*< 0.0001*
BpCr4 - Identify place of delivery/skilled attendant	0.63	*< 0.0001*
BpCr5 - Knows who the blood donor is	1.72	*< 0.0001*
Knowledge on labor and delivery danger signs		*Cronbatch alpha = 0.89*
DsK2 - Vaginal bleeding	1	
DsK3 - Convulsion	1.73	*< 0.0001*
DsK4 - High Fever	1.53	*< 0.0001*
DsK5 - Loss of Consciousness	1.17	*< 0.0001*
DsK6 - Retained Placenta	0.68	*< 0.0001*
DsK7 - Prolonged labor	1.31	*< 0.0001*
Family Support		*Cronbatch alpha = 0.85*
Fs1 - Provided advise/support to deliver at health facilities	1	
Fs2 - Help to get emergency transport during labor	1.04	*< 0.0001*
Fs3 - Accompany during labor	2.31	*< 0.0001*
Fs5 - Help to attend post-natal visits	0.19	*< 0.0001*

**Fig. 4:**
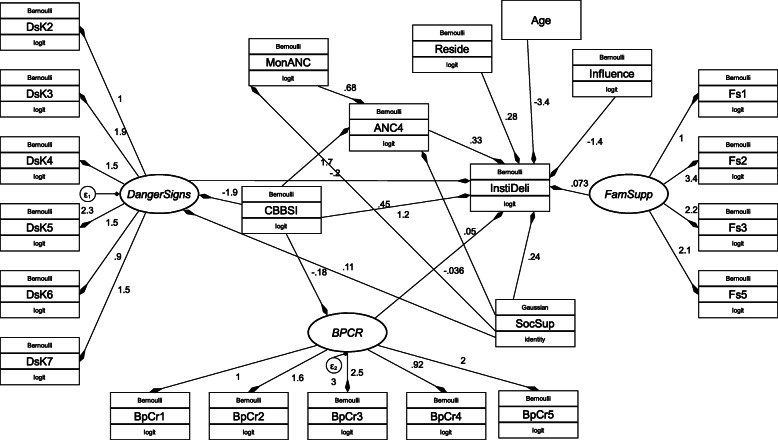
Unstandardized parameter estimates of pathways from CBBS Intervention to institutional delivery, generalized structural equation modeling, January 2019-September 2020, Northwest Ethiopia, (DkS: Danger sign Knowledge, BPCR: Birth preparedness and complication readiness, Famsup: Family support, InstiDeli: Institutional delivery, Danger sign: Knowledge of labor and delivery danger signs, SocSupp: Social support, Fs: Family support)

The estimated path coefficients from the generalized structural equation model were showed in Fig. 4. In addition, path coefficients along with their *p*-values and confidence intervals were indicated in Table 4. Though the magnitude varies across paths, the finding of this study showed that the intervention was linked both through direct and indirect paths to institutional delivery.

**Table 4:** Adjusted odds ratios from the generalized structural equation modeling, January 2019-September 2020, Northwest Ethiopia

Structural model	AOR	(95%CI)
Outcome: Institutional delivery		
Checklist based box system intervention		
Control (Ref)	–	–
Intervention	3.32*	2.36– 4.66
ANC four visit		
No (Ref)	–	–
Yes	1.39*	1.02 – 1.92
Danger Signs	0.83	0.63 -1.08
BPCRp	0.96	0.78 – 1.19
Social Support	1.27*	1.15 – 1.40
Family Support	1.07	0.93 – 1.25
Influence		
No (Ref)	–	–
Yes	1.28*	1.16 - 1.54
Age	0.03*	0.01 – 0.09
Place of Residence		
Rural (Ref)	–	–
Urban	1.32	0.80 – 2.16
Indirect path: Antenatal care 4 attendance		
Control (Ref)	–	–
Intervention	5.64*	4.18 – 7.61
Month of ANC Initiation		
> 16 weeks of gestation (Ref)	–	–
≤ 16 weeks of gestation	1.97*	1.43 – 2.69
Social support	1.05	0.97 – 1.14
Indirect path: BPCRp		
Control (Ref)	–	–
Intervention	0.84	0.70 – 1.04
Indirect path: Danger signs		
Control (Ref)	–	–
Intervention	0.15	0.11 – 5.05
Indirect Path: Month of ANC Initiation		
Social Support	1.57*	1.45 – 1.69
Indirect path: Danger signs		
Social Support	1.12*	1.09 – 1.14

*ID* institutional delivery, *CBBSI* Checklist based box system intervention, *BPCR* Birth preparedness and complication readiness

*p < 0.05

Of the significant direct paths estimated, checklist based box system intervention accounted higher than the other path coefficients that are linked to institutional delivery, (AOR = 3.32, 95%CI: 2.36-4.66) followed by antenatal care four visits (AOR = 1.39, 95%CI: 1.02-1.92) and social supports (AOR = 1.27, 95%CI: 1.15-1.40).

However, from the direct paths, age of the mother (AOR = 0.03, 95%CI: 0.01-0.09) and influence of significant others (mother, grandmother, mother, neighbors, and husband) on the process of using maternal health services (AOR = 1.28, 95%CI: 1.16-1.54) showed an inverse relationship with institutional delivery.

Indirect paths to institutional delivery from the intervention through BPCRp, knowledge of danger signs of labor and delivery, month of ANC initiation, and ANC four visits were created. Similarly, social support was linked by indirect path links through month of ANC initiation and ANC four visits to institutional delivery. Of this indirect path links, the path from the intervention to antenatal care four was the highest significant path (AOR = 5.64, 95%CI: 4.18-7.61), followed by the path from the month of ANC initiation to ANC 4 (AOR = 1.97, 95%CI: 1.43-2.69). The indirect path from social support to month of ANC initiation (AOR = 1.05, 95%CI: 0.97-1.14) and also from social support to knowledge of danger signs of labor and delivery (AOR = 1.12, 95%CI: 1.09-1.14) turned significant (Fig. 4, Table 4).

The total effect of checklist based box system intervention on institutional delivery was 5.92 (AOR = 5.92, 95%CI: 3.34-10.38). The direct component of this effect was3.32 (AOR = 3.32, 95%CI: 2.36-4.66). In addition, ANC 4 visit was found to be a mediator, 33% of the effect of the intervention on institutional delivery was partially mediated by antenatal care four attendance (Table 5).

**Table 5:** Direct, indirect and total effect of CBBSI on institutional delivery, January 2019-September 2020, Northwest Ethiopia

Outcome: institutional delivery		AOR (95% CI)	*p*-value
CBBS Intervention			
Total effect		5.92 (3.34 – 10.38)	< 0.001
Direct effect		3.32 (2.36 – 4.66)	< 0.001
Indirect effects via	Antenatal Care Four attendance	1.79 (1.02 – 3.13)	0.04
	Knowledge of Labor and delivery danger signs	1.45 (0.87 – 2.43)	0.89
	Birth preparedness and complication readiness practice	1.01 (0.97 – 1.04)	0.75

## Discussion

The result of this study showed that checklist-based box system intervention was found effective in improving institutional delivery. In addition, the pathways through which the intervention was linked were tested. Accordingly, the pathway between the intervention and institutional delivery was mainly direct. However, antenatal care four visit partially mediated the effect. The remaining indirect pathways turned insignificant. This study has a similar finding with other studies which showed the contribution of four antenatal care to institutional delivery [14, 15, 22–24, 27]. When the frequency of contact between mothers and health care providers is increasing, health messages delivered during counseling and health education sessions increased and mothers will get enough time to internalize the messages delivered by health care providers. Though most of the association between the intervention and institutional delivery was direct, antenatal care four attendance was found a mediator trough the path to institutional delivery. This could indicate the importance of the continuum of care approach while designing maternal health interventions.

In Ethiopia, m-health interventions and educational messages were used in one of the interventional study aimed at improving maternal health utilization through a continuum of care approach [19]. The other two interventions aimed to increase institutional delivery utilization, the first by improving maternity waiting home utilization [18] and the second by deploying trained community-based nurses to rural communities [20]. The former was implemented m-health interventions in order to increase maternal health service utilization (ANC, institutional delivery and PNC). However, most mothers, particularly in rural Ethiopia, did not own mobile phones. Using CBBSI could be beneficial in this regard because it does not necessitate infrastructure, telecom networking, or the ownership of mobile phones. The latter two interventions, which focus solely on improving institutional delivery, didn’t take the continuum of care approach into consideration.

In this study, there was no observed effect of the intervention through birth preparedness and complication readiness practice and knowledge of labor and delivery danger signs. However, there are studies that showed, the positive effect of both birth preparedness practice [31] and knowledge of labor and delivery danger signs on utilization of institutional delivery [8, 29]. These discrepancies might be because of the nature and approaches of the study and difference while applying the measurement tools. This intervention has both demand creation and service utilization monitoring components. The above finding could indicate that, service utilization component of the intervention, which was implemented through drop-out tracing mechanisms, contributed more for institutional delivery through increasing ANC 4 attendances than the demand creation component. In addition, this study measured BPCR practice of mothers, not the knowledge component. The intervention might have an effect on improving the knowledge of mothers on things that should be fulfilled while preparing for labor and delivery, but the practice is always considered as the effort of mothers.

Social support was found to have a positive and significant relation with knowledge of danger signs of labor and delivery. Similarly, the study showed a positive and significant relationship between social support and institutional delivery. This is in agreement with a study conducted in Kersa, Ethiopia [24], which recommend the importance of social support structures for improving institutional delivery. In addition, an indirect effect of social support was observed through improving early initiation of antenatal care (before 16 weeks of gestation). This in turn improves frequency of ANC visits. Hence, the use of community level social structures would have a paramount importance in disseminating health related messages. The intervention could also be benefited, if the community level demand creation activities could be reached through an identified and functional social structures.

Other determinants of maternal health service utilization, such as age and influence of significant others have also showed a direct but inverse relation with institutional delivery. In the study area, going to health facilities for childbirth service was considered as a lack of internal strength of a woman, especially by older generation of individuals (mother, mother in-law, grandmother and husband) who probably influence the decision of a mother. So, extending demand creation activities to reach to significant others, who might have a decisive role on the life of a mothers could contribute indirectly.

## Strength and limitation

The study used a double-blind approach, in which mothers and outcome assessors were unaware of the intervention, which groups belonged to the intervention and control groups, and the hypothesis that would be tested. In these process, the study limits the occurrence of ascertainment bias or detection bias. Furthermore, during analysis of the effect size, the study’s use of simple randomization procedures was taken into account by employing difference-in-difference estimation. However, because the utilization of maternal health services was self-reported, there is a possibility of self-reporting or recall bias as a limitation.

### Sustainability

The checklist-based box system intervention was built on the government’s existing health system structure, in which health posts are linked to nearby health centers. In addition, the intervention was carried out using the country’s government-owned flagship program, the health extension program (which is being implemented throughout the country). In this regard, the authors believe that the intervention has a high likelihood of being sustainable. However, more research on the feasibility, sustainability, and adaptability of the intervention is needed.

## Conclusion

Checklist based box system intervention was found effective in improving institutional delivery. The pathway through which the intervention was linked with institutional delivery was mainly direct, however, it was partially mediated by antenatal care four visits. In addition, social support and month of initiation of ANC showed a positive relation with institutional delivery. In the contrary, age and influence of significant other inversely related with institutional delivery. The implementation of checklist based box system intervention need to consider the synergetic and complementary effects of maternal health services on each other. Also, this intervention could be re-designed to incorporate functional community level structures for demand creation activities.

## Supplementary Information

**Additional file 1.**

**Additional file 2.**

## Data Availability

The datasets used and/or analyzed during the current study are available from the corresponding author on reasonable request.

## Abbreviations

ANC: Antenatal care
BPCRp: Birth preparedness and complication readiness practice
CBBSI: Checklist based box system intervention
CFA: Confirmatory factor analysis
DsK: Danger signs Knowledge
ID: Institutional delivery
PHCU: Primary health care unit
GSEM: Generalized structural equation modeling
