# Supplementary material for: Effect of checklist based box system interventions on improving institutional delivery among reproductive age women in Northwest Ethiopia: generalized structural equation modeling
Source: Arch Public Health. 2022 Jan 4;80:5. doi: 10.1186/s13690-021-00774-2 (PMC8725524; doi:10.1186/s13690-021-00774-2)
Supplement: Supplementary file 1 — Additional file 1. [file 13690_2021_774_MOESM1_ESM.docx]

**Effectiveness of checklist based box system interventions versus routine care on improving maternal health care Utilization in Northwest Ethiopia: A cluster randomized controlled trial**

**Study area: Northwest, Ethiopia**

**Questionaries’ for mothers who delivered one year before the survey**

**Participant Information Sheet**

**Research Project Title**

**Effectiveness of Checklist-Based Box System Interventions (CBBSI) versus routine care on improving maternal health Care Utilization in Northwest Ethiopia: A cluster randomized controlled trial**

**Introduction**

Good morning/afternoon! My name is _________________. I am the member of the research team from Jimma University. We are speaking with mothers about their experience during pregnancy, delivery and the post-partum period.

**Purpose of the Research**

This research aims to investigate the effectiveness of checklist based box system on improving utilization of maternal health services antenatal care, health facility delivery and post-natal care. This research will investigate the effects of the aforementioned intervention of person centered health education at community and health post level and monitoring box implementation at health center on the improvement of service utilization.

**Research procedure**

We have selected mothers to participate in this study by chance. You are among those selected mothers to be part of this study. I will ask you questions concerning personal, socio-demographic and your experience during your last pregnancy, delivery and the post-partum period. I will also ask the information you have regarding the knowledge and health service seeking behavior you had during your last pregnancy. The interview will take about 40 minutes. So, I kindly request your volunteer participation for this study.

**Possible Disadvantage and Risks**

Participating in this research is not anticipated to cause you any disadvantages or discomfort. If you feel discomfort sharing personal information, you can refuse answering.

**Benefit**

Whilst there are no immediate benefits for those people participating in the project, it is hoped that this work will have a beneficial impact on knowing the effectiveness of the aforementioned intervention on its contribution of improving utilization of maternal health services.

**Confidentiality**

All the information that we collect about you during the course of the research will be kept strictly confidential. You will not be able to be identified or identifiable in any reports or publications. Any data collected about you in the data collection device will be stored online in a form protected by passwords. Data collected may be shared in an anonymised form by the research team. These anonymised data will not allow any individuals or their institutions to be identified or identifiable.

**Refusal/Withdrawal**

If you are not comfortable, you can refuse to answer any question. You may stop participating in the interview at any time if not convenient for you without losing any of your rights as a participant. However, your active participation and genuine responses have paramount importance in improving maternal health services in future.

**Informed Consent Agreement Form**

**English Version**

Good Morning/Good Afternoon: My name is (name of the data collector). I kindly invited you to participate in the study investigating the effectiveness of checklist based box system on maternal health service utilization improvement. This study is conducted by Jimma University. The finding of the study will be used as an input for the project assessing the effectiveness of checklist based box system implementation on the improvement of maternal health service utilization. You will not get any risk by participating on this study, but your response together with the response of others will be used as an input to for the project aiming to improve the utilization of maternal health services. If you don’t want to continue with the responding questions, you may refuse to answer questions at any time. If you need further information about the study, you can contact a representative of the research team, Ms. Netsanet Belete (+251-938-935135)

With due understanding of the aforementioned information, are you willing to participate in the study? **Yes, Continue No, End**

**Study Tool**

| **Questionnaire Prepared For Mothers Delivered In the last one Year (12 Months)** |
| --- |

| **Part I – Educational and Ecological Assessment** | | | |
| --- | --- | --- | --- |
| Identification Code: ______________  Name of Woreda:_______________  Name of Kebele:________________  Surrounding Health Center:________________  Data Collectors Code:_______  Supervisors Code:_______ | | | |
| **S.no** | **Question** | **Response Category** | **Skip** |
| 101 | Age | ___________ Years |  |
| 102 | Where is your current residency? | Urban……………………..1  Rural……………………....0 |  |
| 103 | Did you attend school? | Yes………………………...1  No…………………………0 | No =>105 |
| 104 | Can tell me your level of education? | Can’t read and write………………1  Non-formal education……………..2  Formal education (1-8^th^ grade)…...3  Formal education (9-12^th^ grade)….4  Above 12^th^ grade………………...5  I don’t know……………………88  Others, Specify _____________99 |  |
| 105 | Religion | Orthodox Christian………………..1  Muslim…………………………….2  Protestant………………………….3  Catholic……………………………4  Others, Specify_______________99 |  |
| 106 | Ethnicity | Amhara…………………………..1  Oromo……………………………2  Tigre……………………………....3  Guragie…………………………...4  Others, Specify______________99 |  |
| 107 | Occupation | Government Employee…………..1  Private…………………………….2  Merchant…………………………3  Student…………………………....4  Farmer……………………………5  House Wife……………………….6  Others, Specify_______________99 |  |
| 108 | Marital Status | Single……………………………..1  Married…………………………..2  Divorced…………………………3  Husband not alive……………….4 |  |
| 109 | Age at first delivery | ___________ Years |  |

**Wealth Index**

| **S.no** | **Question** | **Response Category** | **Skip** |
| --- | --- | --- | --- |
| 110 | How many members in your family?  **(Family member is those who eat from the same meal)** | ___________ |  |
| 111 | What is the main source of drinking water for your family? | Pipe water  Within the compound……………...11  Out of the compound ……………...12  Bono………………………………..13  Head water  Protected head water……………….21  Unprotected head water……………22  Pond  Protected Pond……………………..31  Unprotected Pond………………….32  Well  Within the compound……………..41  Common well……………………...42  Ground water  Lake/River/Beck/Dam…………….51  Rain water………………………….61  Tanker……………………………...71  Packed water……………………….91  No regular source of water………...96  Others, Specify_______________­­_99 |  |
| 112 | What time did it require to and come back to this common source of water? (in Minutes) | ­­­­­­­____________ Minutes |  |
| 113 | What did you do, to make this water ready for drinking?  ***Interviewer: Multiple answer is possible)*** | Boiling……………………………..1  Chlorine…………………………....2  Filtering……………………………3  By using sand……………………..4  Using sunshine……………………5  Keep the water to filter by itself…..6  I don’t know………………………88  Others, Specify_______________­­_99 |  |
| 114 | What type of Latrine do this family is using?  **(Interviewer: One response is possible. If the response is more than one, please select the one which is approximate with the listed one). Observe the Toilet** | Flash Latrine  Proceeding to septic tank…………11  Proceeding to pit latrine…………..12  Proceeding to unknown Place…….13  Pit Latrine  Traditional Pit latrine……………..21  Traditional pit latrine by cement…22  Traditional pit latrine not by cement…………………………….23  Ventilated Pit Latrine……………..24  Bucket/Bedpan……………………25  Compost Latrine…………………..26  Forest/field………………………...31  Others, Specify_______________­­_99 |  |
| 115 | Did anyone, other than your family, share this latrine? | Yes………………………...1  No…………………………0 |  |
| 116 | How many households are using this latrine, including your household? | ____________ | No =>117 |
| 117 | What power source does your family use for cooking?  ***(Interviewer: Multiple Responses are Possible)*** | Electricity………………………….1  Natural gas………………………...2  Biogas……………………………...3  Kerosene…………………………...4  Charcoal…………………………...5  Wood………………………………6  Grass………………………………7  Ruminants…………………………8  Muck……………………………….9  No cooking in this house…………10  Others, Specify_______________­­_99 |  |
| 118 | Where do you usually cook? | In side Home………………………..1  Separated home…………………….2  Outside………………………………3  Others, Specify_______________­­_99 |  |
| 119 | Did you have separated room for cooking? | Yes………………………...1  No…………………………0 |  |
| 120 | How many bedrooms do you have in your house? | ______________ Rooms |  |
| 121 | Are there domestic animals, cows, and animals for farming purposes or hens found in this house? | Yes………………………...1  No…………………………0 | No =>123 |
| 122 | What types of domestic animals do you have in this house?  ***(Interviewer: Multiple Responses are Possible)*** | Milking cow, ox, bull………………1  Other cattle………………………....2  Hoarse, Donkey, Mule……………..3  Camel………………………………4  Goat………………………………...5  Sheep……………………………….6  Hen…………………………………7  Beehive……………………………..8 |  |
| 123 | Do you/member of your family own farming land? | Yes………………………...1  No…………………………0 | No =>125 |
| 124 | How many hectares of farming land do members of this family own?  ***(Interviewer: Please change the local measurement (Timad) in to Hectare (1 Hectare = 4 Timad)*** | ___________ Hectare |  |
| 125 | Can you please tell me which one of the following is in your house?  ***(Interviewer: Multiple Responses are Possible)***  Electricity……………………  Radio………………………..  Television…………………...  Fixed phone…………………  Computer……………………  Refrigerator………………….  Table…………………………  Chair…………………………  Bed with Mattress……………  Electric Mitad………………..  Kerosene Power……………… | Electricity…………………………1  Radio………………….…………..2  Television……………….………...3  Fixed phone………………………4  Computer…………………………5  Refrigerator……………………….6  Table………………………………7  Chair………………………………8  Bed with Mattress……..….………9  Electric Mitad……………..……..10  Kerosene Power…………..………11 |  |
| 126 | Do members of your family own one of the following?  ***(Interviewer: Multiple Responses are Possible)***  Watch…………………………..  Mobile………………………….  Bicycle……………………….....  Motor Cycle……………………  Cart…………………………….  Car……………………………..  Bajaj…………………………… | Watch…………………………....1  Mobile…………………………....2  Bicycle……………………….......3  Motor Cycle…………………..…4  Cart……………………………....5  Car……………………………….6  Bajaj…………………………..…7 |  |
| 127 | From which material do the floor of your house was made? | Smirch………………………………1  Wood…………………………….....2  Cement…………………………......3  Others, Specify_______________­­_99 |  |
| 128 | From which material do the wall of your house was made? | Smirch………………………………1  Wood…………………………….....2  Cement…………………………......3  Others, Specify_______________­­_99 |  |
| 129 | Did you use iodized salt for your meals? | Yes………………………...1  No…………………………0 |  |

| **Part II Pregnancy, Delivery and Postnatal Care Related Questions**  *(Now I will ask you questions related to pregnancy, delivery and postnatal care)* | | | |
| --- | --- | --- | --- |
| **2.1 Knowledge Related Questions** | | | |
| **S.no** | **Question** | **Response Category** | **Skip** |
| 201 | How many Pregnancies did you have?  ***(This includes abortions)*** | ___________ Times |  |
| 202 | How many deliveries did you have?  ***(Even though she/he is alive for minutes/hours, if births/crying was detected)*** | ___________ Times |  |
| 203 | How many alive under five children do you have? (Male and Female) | _______ Male (Under Five)  _______Female (Under Five)  _______Total (Under Five) |  |
| 204 | Do you think pregnant women should have a regular antenatal care follow-up during her pregnancy? | Yes………………………...1  No…………………………0 | No =>206 |
| 205 | If your answer for question 204 is ‘Yes’ do you think a pregnant mother should go for antenatal care follow-ups, if there are no problems and illnesses? | Yes………………………...1  No…………………………0 |  |
| 206 | When do you think is the time that a pregnant woman should start antenatal follow-up? | Within the first three months……..1  Within the first five months………2  I don’t know……………………...88  Others, Specify_______________99 |  |
| 207 | How many antenatal follow-ups do a pregnant woman should have throughout her pregnancy? | 1-3 times………………………….1  Four and above…………………..2  I don’t know……………………...88  Others, Specify_______________99 |  |
| 208 | Do you think a pregnant woman should take TT vaccination during her pregnancy? | Yes………………………...1  No…………………………0 | No =>210 |
| 209 | If your answer for question 208 is ‘Yes’, how many TT vaccinations would be enough for a pregnant women for a single pregnancy? | One time………………………...1  Two times………………………2  Three times……………………..3  Four times………………………4  Five times………………………5  I don’t know……………………88  Others, Specify______________99 |  |
| 210 | Do you think a pregnant woman should take Iron folate to prevent anemia? | Yes………………………...1  No…………………………0 |  |
| 211 | DO you think a pregnant woman should take additional food during her pregnancy? | Yes………………………...1  No…………………………0 |  |
| 212 | What do you think are the benefits of attending ANC?  ***(Interviewer: Multiple responses Possible)*** | To know the health of the mother….1  To know the condition of baby ....….2  Early detection and treatment of problems...…………………………..3  Prevention of complications and disease…………………………...…..4  Birth Preparedness…………….…….5 To know and detect danger signs of pregnancy…………………………...6 For safe delivery…………………….7  I don’t know……………………….88  Others, Specify________________99 |  |
| 213 | Do you know danger signs of pregnancy?  ***(Interviewer: Multiple responses Possible)*** | Vaginal bleeding..................................1 Sudden gush of fluid or leaking of  fluid from vagina.................................2 Severe headache not relieved by  simple analgesics.................................3 Dizziness and blurring of vision.........4 Sustained vomiting..............................5 Swelling (hands, face, etc.).................6 Loss of fetal movements.....................7 Convulsions..........................................8 Premature onset of contractions  (before 37 weeks).................................9 Severe or unusual abdominal pain....10 Chills or fever....................................11  I don’t know………….…………......88  Others, Specify_________________99 |  |
| 214 | What are the things that you prepared for your last delivery?  ***(Interviewer: Multiple responses Possible)*** | Transportation in case of emergency…………………………...1 Emergency funds.................................2 Personal savings and how to access it  in case of need.....................................3 Chooses skilled attendant/place of  birth...................................................4  Knows who the blood donor is……..5  I don’t know………….…………......88  Others, Specify_________________99 |  |
| 215 | What are dangers Signs during labor and childbirth?  ***(Interviewer: Multiple responses Possible)*** | Severe headache ……………..……...1  Vaginal bleeding …………………….2  Convulsions……………………...….3  High fever……………………….….4  Loss of consciousness………….…...5  Prolonged labor……………….……6  Retained placenta……………..……7  I don’t know………….………….....88  Others, Specify_________________99 |  |
| 216 | What do you think are the benefits of delivering at health institutions attended by skilled health care providers?  ***(Interviewer: Multiple responses Possible)*** | Provide care for women/fetus during  labor and delivery………............…..1  Provide care for women/fetus during  the immediate postpartum period…2  Provide care for complications during  labor and delivery…...........................3  Detect and manage complications  during the immediate postpartum period..................................................4 Provide emergency management in  case of emergency…..........................5  Provide pre-referral management in  cases of emergency..............................6  I don’t know………….…………......88  Others, Specify________________99 |  |
| 217 | How many times does a mother should follow for postnatal care after delivery? | 1-2 times …………………………..1  3 times ………………………...…...2  I don’t know………….………….....88  Others, Specify________________99 |  |
| 218 | What do you think are the benefits of postnatal care?  ***(Interviewer: Multiple responses Possible)*** | Early detection and management of complications………………..……..1 Promoting health and preventing disease………………….………......2 Providing woman-centered education  and counselling……………....……..3  I don’t know………….………….....88  Others, Specify________________99 |  |
| 219 | What do you think are the benefits of postnatal care?  ***(Interviewer: Multiple responses Possible)*** | Sever vaginal bleeding…………..…1  Severe headache………………..….2  Blurred vision………………..……..3  Convulsions………………………...4  Swollen hands/face……….………..5  High fever…………...……………...6  Loss of consciousness………..…….7  Difficulty breathing……….………..8  Sever weakness……………………..9  Foul smelling vaginal discharge…..10  I don’t know………….………….....88  Others, Specify________________99 |  |

| **2.2 Social and Epidemiological Assessment** | | | |
| --- | --- | --- | --- |
| **S.no** | **Question** | **Response Category** | **Skip** |
| 301 | Did you have ANC follow up for your last baby? | Yes………………………...1  No…………………………0 | No =>308 |
| 302 | How many times did you go to health facilities for your ANC follow-up? | __________ |  |
| 303 | At what gestational age was your first visit? | __________ Months |  |
| 304 | Did you experience health talks in your ANC follow-ups? | Yes………………………...1  No…………………………0 |  |
| 305 | Where was your antenatal care follow-up for your last pregnancy? | Government Health post……….1  At home by HEWs……………...2  Government health center……...3  Government hospital…………...4  Private Hospital………………...5  Private Clinic…………………....6  At home by TBAs………………7  I don’t know………….………...88  Others, Specify_____________99 |  |
| 306 | How much time did it take to reach to the nearby health facility? | ___________ Minutes |  |
| 307 | Who provide you advice about antenatal care follow-up (benefits) for your last pregnancy? | Health center/Hospital Professionals..1  Health Extension Workers…………..2  TBAs………………………………...3  Relative/friends…………………….4  1-5 Model Family…………………...5  Other Family members……………..6  My Husband………………………...7  I don’t know………….………..…...88  Others, Specify_____________­­­­­___99 |  |
| 308 | What was the reason for not attending Antenatal care?  ***(Interviewer: Multiple responses Possible)*** | I don’t know the complications of pregnancy............................................1 I don’t know the benefits of attending antenatal care.......................................2  I don’t know when to start ANC.........3 I don’t know about danger signs of pregnancy............................................4  I don’t know when to go and how  many times to go……………….…...5  Autonomy/decision making power....6  Wontedness of pregnancy ..................7 Ever faced pregnancy related complications......................................8 Lack of previous maternal health  follow-ups...........................................9  opposition of husband.......................10  Not feeling sick ................................11 Workload...........................................12 Poor quality of care...........................13 Facility not opened............................14 Didn’t experience compassionate  and respectful care………..……….15 Long waiting time............................16 Health professionals didn’t schedule when to come……………………...17  I don’t know………….………..…...88  Others, Specify_____________­­­­­___99 |  |
| 309 | What was the average amount of time that you waited to see medical staff when you visited the health facility? | Less than 30 min…………………..1  30 min to <1and ½ hours…….……2  1 and ½ hours to 2 hours…………..3  More than 2 hours…………….…...4  I don’t remember………………….5 |  |
| 310 | What health services did you receive when you visited the clinic during your pregnancy?  ***(Interviewer: Multiple responses Possible)*** | Physical examination (including  weight, blood pressure, heart rate)...1  Gynaecologic examination…….….2  Ultrasound…………………………3  HIV/STD testing……………...……4  Blood test…………………………..5  Nutritional supplements……..…….6  Tetanus Vaccine………...…………7  I don’t remember…………………..8 |  |
| 311 | Did you attend four plus ANC follow-up for your last baby? | Yes………………………...1  No…………………………0 | Yes=>313 |
| 312 | If your answer for question 311 is ‘No’, What was the reason/s for not attending four plus ANC?  ***(Interviewer: Multiple responses Possible)*** | I don’t know the complications of pregnancy...........................................1 I don’t know the benefits of attending  full ANC antenatal care......................2   I don’t know when to go and how  many times to go……………………3  Autonomy/decision making power....4  Wontedness of pregnancy ..................5 Ever faced pregnancy related complications.......................................6 opposition of husband.........................7 Not feeling sick………………….......8 Workload.............................................9 Poor quality of care...........................10  Facility not opened...........................11 Didn’t experience compassionate  and respectful care……………...….12  Long waiting time............................13 Health professionals didn’t schedule when to come....................................14 Place of residence.............................15  I don’t know………….………..…...88  Others, Specify_____________­­­­­___99 |  |
| 313 | Were any complications detected during your pregnancy period? | Yes………………………...1  No…………………………0 | No=>317 |
| 314 | Did the primary clinic provide emergency care for these complications? | Yes………………………...1  No…………………………0 | Yes=>317 |
| 315 | What was the primary reason you did not receive emergency care for complications?  ***(Interviewer: Multiple responses Possible)*** | No skilled birth attendant………….1  Necessary drugs unavailable………2  Necessary medical…………………3  Supplies/equipment unavailable……4  No transport to secondary hospital…5  I don’t know………….………..…...88  Others, Specify_____________­­­­­___99 |  |
| 316 | Were you referred to a secondary hospital for treatment of these complications? | Yes………………………...1  No…………………………0 |  |
| 317 | Where did you gave birth for your last pregnancy? | Home …………………………...….1  Health Center………………………2  Government Hospital………….…...3  Private Hospital………………….…4 | Home=> 323 |
| 318 | Did your child alive? | Yes………………………...1  No…………………………0 |  |
| 319 | What is the sex of (Name)? | Male………………………...1  Female……………………...2 |  |
| 320 | Age of (name)? | ___________ Months |  |
| 321 | What was the average amount of time that you waited to see medical staff when you visited the health facility? | Less than 30 min…………………..1  30 min to <1and ½ hours…….……2  1 and ½ hours to 2 hours…………..3  More than 2 hours…………….…...4  I don’t remember………………….5 |  |
| 322 | If you gave birth to the last baby at a health facility, why?  ***(Interviewer: Multiple responses Possible)*** | For safe delivery…………….…….1  For healthy child………………..…2  Free Health care……………….…..3  Health facility nearby……………..4  Good health service………………..5  Encouraged by family………….….6  Motivated by health care workers…7 |  |
| 323 | If the answer for question 317 is ‘home’, what was the reason?  ***(Interviewer: Multiple responses Possible)*** | I don’t know complications of  labour and delivery…………...........1  I don’t know benefits of  institutional delivery……………….2 Autonomy/decision making power………………………………..3  Wontedness of pregnancy...................4  Place of residence................................5  Ever-faced labor and delivery  related complications...........................6  Lack of previous maternal health follow-ups…………………………....7  Lack of birth preparedness plan……..8 Labor was too quick…………..….….9  Misunderstanding after being sent to home for latent stage labor................10 Cultural factors..................................11 Poor quality of care...........................12  Facility not opened……….……......13 Health facility too far, didn’t experience ….....................................14 Compassionate and respectful care..15  My neighbour/relative didn’t experience compassionate and respectful care……………………...16 Health professionals don’t allow birth companion.........................................17 Health providers didn’t allow preferred birthing positions...............................18  I don’t know………….………..…...88  Others, Specify_____________­­­­­___99 |  |
| 324 | Were any complications detected during your labor and delivery? | Yes………………………...1  No…………………………0 | No=>328 |
| 325 | Did the primary clinic provide emergency care for these complications? | Yes………………………...1  No…………………………0 | Yes=>328 |
| 326 | What was the primary reason you did not receive emergency care?  ***(Interviewer: Multiple responses Possible)*** | No skilled birth attendant……….....1  Necessary drugs unavailable…....…2  Necessary medical…………..…..…3  Supplies/equipment unavailable…..4  No transport to secondary hospital...5  I don’t know………….………..…...88  Others, Specify_____________­­­­­___99 |  |
| 327 | Were you referred to a secondary hospital for treatment of these complications? | Yes………………………...1  No…………………………0 |  |
| 328 | What do you think are the reasons for not attending health facility delivery?  ***(Interviewer: Multiple responses Possible)*** | Health facility delivery is not important……………………………1  Facility delivery is not a habit………2  My husband/other family member didn’t allow………………………….3  No female health workers at health facilities……………………………...4  I didn’t believe on health by the work of professionals……………………...5  Facility Closed………………………6  Health facility far/No transport…….7  High price related to facility delivery.8  Low quality delivery care……………9  Others, Specify_____________­­­­­___­­_99 |  |
| 329 | Who attended you when you gave birth (name)?  ***(Interviewer: Multiple responses Possible)*** | Health Provider (Doctor, Health Officer, Nurse, Midwife)……………1  Trained TBA………………………...2  Untrained TBA……………………....3  Health Extension Worker…………...4  Relative/friend/neighbor……..……5  No one………………………………6  Others, Specify_____________­­­­­___­­_99 |  |
| 330 | Did you have PNC for your last baby? | Yes………………………...1  No…………………………0 | No=>337 |
| 331 | If your answer for question 330 is ‘yes’ How many times did you go to health facilities for your PNC follow-up? | ____________ |  |
| 332 | When did you get the first PNC after you delivered (name)? | Immediately After delivery………….1  2 days after delivery…………………2  3-7 days after delivery………………3  7-28 days after delivery……………...4  1 month after delivery……………….5 |  |
| 333 | Who attended you for PNC after you delivered (name)?  ***(Interviewer: Multiple responses Possible)*** | Health Professionals………………..1  Traditional birth attendants………...2  Health Extension Workers………….3  Relative/friend/neighbor……..……4  Others, Specify_____________­­­­­___­­_99 |  |
| 334 | Do you/your family report to HEWs after 48 hour of delivery of (name)? | Yes………………………...1  No…………………………0 |  |
| 335 | Did you experience health talks in your PNC follow-ups? | Yes………………………...1  No…………………………0 |  |
| 336 | What health services did you receive when you visited the clinic after your delivery? (Multiple responses possible)? | Physical examination…………….….1  Counselling on breast feeding…….....2  Contraceptives………………………3  Blood test for anemia……………….4  Nutritional supplements…………….5  Information on warning signs of problems…………………………..…6  I don’t remember …………………....7  Others, Specify_____________­­­­­___­­_99 |  |
| 337 | What was your reason for not attending postnatal care?  ***(Interviewer: Multiple responses Possible)*** | I don’t have any complication  during the postnatal period……….…1  I don’t know when to seek care  for post-natal care…………….……2  Postnatal care is only for  immunization of new born………....3  Autonomy/decision making power...4  Wontedness of pregnancy……….....5  Place of residence…………………..6  Lack of previous maternal  health follow-ups………………..….7  Workload ………………….……….8  I don’t want to miss cultural  things at home….………………….9  Birth outcome……………………..10  Poor quality of care………………..11  Facility not opened…….…………..12  Health professionals didn’t  schedule when to come…………....13  I don’t remember …………………..14  Others, Specify_____________­­­­­___­­_99 |  |
| 338 | Were any complications detected during your postnatal period? | Yes………………………...1  No…………………………0 | No=>342 |
| 339 | Did the primary clinic provide emergency care for these complications? | Yes………………………...1  No…………………………0 | Yes=>342 |
| 340 | What was the primary reason you did not receive emergency care?  ***(Interviewer: Multiple responses Possible)*** | No skilled birth attendant………….1  Necessary drugs unavailable………2  Necessary medical supplies/  equipment unavailable..................…3  No transport to secondary hospital...4  I don’t remember …………………..5  Others, Specify_____________­­­­­___­­99 |  |
| 341 | Were you referred to a secondary hospital for treatment of these complications? | Yes………………………...1  No…………………………0 |  |
| 342 | Is there anyone who influences you on the process of using maternal health services? | Yes………………………...1  No…………………………0 | No=>344 |
| 343 | If your answer for question number 342 is ‘Yes’, who influences you?  ***(Interviewer: Multiple responses Possible)*** | Grandmother………………….…..1  Mother……………………………2  Mother in law……………………..3  Husband……………………….….4  Neighbor/Friends……..…………..5  Others, Specify_____________­­­­­___­­99 |  |
| 344 | What do you think are social factors that influence the use of maternal health services in your facility?  ***(Interviewer: Multiple responses Possible)*** | Mother’s autonomy……………...…1  Mother’s decision making power…..2  Husband Refusal…………………...3  Lack of spousal discussion  regarding MHS……………………..4  I don’t remember …………………..5  Others, Specify_____________­­­­­___­­99 |  |
| 345 | What type of transportation do you use in cases of emergencies? | Ambulance…………………………1  Public Transport……………………2  Private Transport…………………...3  Traditional way of transportation….4  I don’t remember …………………..5 |  |
| 346 | How do you see the supply of maternal health equipment and supplies in the health facility you receive MHS for the last time? | Always available…………………...1  Usually available…………………..2  Sometimes available……………….3  Always unavailable………………..4  Usually unavailable………………..5  Sometimes unavailable……………6  I don’t remember ………………….7 |  |
| 347 | Did you get maternal health services free of charge? | Yes, Always………………………..1  Yes, Usually………………………..2  Yes, Sometimes……………………3  Didn’t get free of charge…………...4 |  |

| **2.3 Behavioral and Environmental Assessment** | | | |
| --- | --- | --- | --- |
| **S.no** | **Question** | **Response Category** | **Skip** |
| 401 | Was your last pregnancy wonted? | Yes………………………...1  No…………………………0 |  |
| 402 | Did you have a birth preparedness plan in your last pregnancy? | Yes………………………...1  No…………………………0 | No=>405 |
| 403 | If your answer for Q no. 402 is ‘yes’, who support you to develop this plan? | Myself………………………….…..1  Me with my husband……………....2  Me with health care providers……...3  Me with health Extension Worker....4  Others, Specify_____________­­­­­___­­99 |  |
| 404 | What are the specific plans you focus during that time? | Saved money……………………….1  Identified skilled provider…….……2  Saved emergency fund ………....…..3  Identified place of delivery………....4  Identified facility that works 24 h.…5  Arranged means of transportation.....6  Others, Specify_____________­­­­­___­­99 |  |
| 405 | How many times did you attend pregnant mothers’ conference in your last pregnancy? | I didn’t attend pregnant mothers conference…………………………..1  1-3 times…………………………….2  4-6 times…………………………….3  More than 7 times………..………….4 | 1 or 2=>406 |
| 406 | If the answer for Q. no. 405 is option 1or 2, what was the reason for not attending pregnant mothers conference? | Work load……………..……..………1  It was organized far from my house….2  It was not organized regularly…..…....3  Others, Specify_____________­­­­­___­­99 |  |
| 407 | Did you get the necessary support from health care providers during your pregnancy, natal and post-natal period? | Yes………………………...1  No…………………………0 | No=>412 |
| 408 | If your answer for Q. no. 407 is ‘yes’ How do you explain the care you got during your pregnancy?  ***(Interviewer: Multiple responses Possible)*** | Got different health interventions…..1  Advise on different health aspects.…2  Advise on different home care……...3  Advise to attend ANC regularly……..4  Advise on danger signs of pregnancy.5  I don’t remember…………………….6 |  |
| 409 | How do you explain the care you got during your natal period?  ***(Interviewer: Multiple responses Possible)*** | Got different health interventions……...1  Advise on different health aspects…….2  Advise on danger signs………………...3  I don’t remember………………...…….4  I didn’t go to health facility during this period…………………………….…….5 |  |
| 410 | How do you explain the care you got during your post-natal period?  ***(Interviewer: Multiple responses Possible)*** | Got different health interventions………1  Advise on danger signs…………………2  Advise on nutrition, BF, FP, Hygiene….3  Advise on how to care my baby…..….4  I don’t remember…………………...…5  I didn’t go to health facility during this period………………………………….6 |  |
| 411 | How do you explain the approach of health care providers during your last MHS follow-ups?  ***(Interviewer: Multiple responses Possible)*** | Approached in a compassionate and  respectful way…………………….…..1  Physical Abuse……………………….2  Detention………………………..…….3  Non-confidential care………………...4  Non-consented care……………...…...5  Abandonment/Neglect………..………6  Non-dignified care………………..…..7  I don’t remember……………..………8 |  |
| 412 | Did you get the necessary support from your family members during your pregnancy period? | Yes………………………...1  No…………………………0 | No=>414 |
| 413 | If your answer for Q no.412 is ‘yes’, can you explain the support you got?  ***(Interviewer: Multiple responses Possible)*** | Help to increase my nutritional in-take..1  Accompany during my follow-up periods……………………………….....2  Help to take rest……………….....…....3  Help to attend health Education session..4  Took the autonomy to decide when I want to go to health facilities………………..5  I don’t remember …………………..….6  Others, Specify_____________­­­­­______­­99 |  |
| 414 | Did you get the necessary support from your family members during your intra-natal and postnatal period? | Yes………………………...1  No…………………………0 | No=>416 |
| 415 | If your answer for Q no. 414 is ‘yes’, can you explain the support you got?  ***(Interviewer: Multiple responses Possible)*** | Provide advice to deliver in Health facility……………………………...…1  Help to get emergency transport  During labor……………………..……2  Accompany during labor………..……3  Help to care my baby………….…..…4  Help to attend post- natal visits…..…..5  I don’t remember …………………….6  Others, Specify_____________­­­­­_____­­99 |  |
| 416 | What do you think are the reasons, raised in our community, for not attending ANC as recommended? (Individual Level)  ***(Interviewer: Multiple responses Possible)*** | Autonomy/decision making power  to go to health institution……………...1  Wontedness of pregnancy……...……..2  Place of residence……………….…….3  Ever faced pregnancy  related complications……………...….4  Lack of previous MH follow-ups……..5  Opposition of husband………………..6  Lack of inter-spousal discussion……...7  Afraid of user fee…………………......8  Cultural factors…………………….…9  I don’t remember ……………..….….10  Others, Specify_____________­­­­­_____­­99 |  |
| 417 | What do you think are the reasons, raised in our community, for not attending ANC as recommended? (Facility Level)  ***(Interviewer: Multiple responses Possible)*** | Poor quality of care………………..…1  Facility not opened…………….……..2  Health facility too far…………………3  Didn’t experience compassionate  and respectful care………………...….4  Long waiting time……………...……..5  Health professionals didn’t  schedule when to come………..….…..6  I don’t remember ……………..….…...7  Others, Specify_____________­­­­­_____­­99 |  |
| 418 | What do you think are the reasons, raised in our community, for not attending facility delivery?  (Individual Level)  ***(Interviewer: Multiple responses Possible)*** | Autonomy/decision making power  to go for institutional delivery………...1  Wontedness of pregnancy………….....2  Place of residence…………………….3  Ever faced labor and  delivery related complications…….….4  Lack of previous MH  follow-ups….........................................5  Opposition of husband……….……...6  Lack of inter-spousal discussion…......7  Afraid of user fee……………………..8  Lack of birth preparedness plan……..9  Labor was too quick…………...…….10  Misunderstanding after being sent to  home for latent stage labor…………..11  Cultural factors………………………12  I don’t remember ……………..….….13  Others, Specify_____________­­­­­_____­­99 |  |
| 419 | What do you think are the reasons, raised in our community, for not attending facility delivery?  (Facility Level)  ***(Interviewer: Multiple responses Possible)*** | Poor quality of care…………..………1  Facility not opened………………..….2  Health facility too far……………..…3  Didn’t experience compassionate  and respectful care…………….……..4  My neighbor/relative didn’t experience  Compassionate and respectful care..…..5  Health professionals didn’t schedule  when to come……………………...….6  Health professionals don’t  allow birth companion…………..…..7  Health providers didn’t allow preferred birthing positions……………..………8  I don’t remember ……………..…...….9  Others, Specify_____________­­­­­_____­­99 |  |
| 420 | What do you think are the reasons, raised in our community, for not attending postnatal care?  (Individual Level)  ***(Interviewer: Multiple responses Possible)*** | Autonomy/decision making power  to go for postpartum/postnatal care……1  Wontedness of pregnancy………..……..2  Place of residence……………….………3  Ever faced postnatal complications……..4  Lack of previous MH follow-ups………..5  Opposition of husband………………….6  Lack of inter-spousal discussion….…......7  Afraid of user fee ……………………….8  Workload………………………….……..9  I don’t want to miss cultural things done  at home level immediately after delivery………………………………...10  Mode of delivery……………….…..….11  Place of delivery……………………….12  Birth outcome……………...…………..13  I don’t remember ……………..…...…...14  Others, Specify_____________­­­­­_____­­__99 |  |
| 421 | What do you think are the reasons, raised in our community, for not attending postnatal care?  (Facility Level)  ***(Interviewer: Multiple responses Possible)*** | Poor quality of care…………………….1  Facility not opened……………………..2  Health facility too far…………….……..3  I didn’t experience compassionate and  respectful care…………….………..…..4  Health professionals didn’t schedule  when to come…………………….……..5  I don’t remember ……………..…...…...14  Others, Specify_____________­­­­­_____­­__99 |  |
| 422 | Did you go to health facilities before your pregnancy for health service or advice? | Yes………………………...1  No…………………………0 |  |
| 423 | How do you the general status of your health? | Very poor…………………………1  Poor……………………………….2  Good……………………………....3  Very good………………………....4 |  |
| 424 | Do you think health facilities in your catchment are enough to deliver maternal health services? | Yes………………………...1  No…………………………0 |  |
| 425 | Is ambulance available all the time, when needed? | Yes, always……………………….1  Yes, usually……………………….2  Yes, Sometimes……………….…..3  No………………………………….4 |  |

| **Health Service Quality** | | | |
| --- | --- | --- | --- |
| **S.no** | **Question** | **Response Category** | **Skip** |
| 501 | Did you go to health facility to deliver your last baby? | Yes………………………...1  No…………………………0 | No=> SS |
| 502 | Is the nearby health facility clean and safe? | Strongly disagree…………………...1  Disagree………………………….....2  Neutral ……………………………..3  Agree……………………………….4  Strongly disagree…………………...5 |  |
| 503 | Were health care providers compassionate and respectful? | Strongly disagree…………………...1  Disagree………………………….....2  Neutral ……………………………..3  Agree……………………………….4  Strongly disagree…………………...5 |  |
| 504 | Did you believe on the on the competency of health care providers? | Strongly disagree…………………...1  Disagree………………………….....2  Neutral ……………………………..3  Agree……………………………….4  Strongly disagree…………………...5 |  |
| 505 | How did you rate the advice that heath providers were providing on initiation of breast feeding? | Strongly disagree…………………...1  Disagree………………………….....2  Neutral ……………………………..3  Agree……………………………….4  Strongly disagree…………………...5 |  |
| 506 | How did you rate the advice that heath providers were providing on cord care? | Strongly disagree…………………...1  Disagree………………………….....2  Neutral ……………………………..3  Agree……………………………….4  Strongly disagree…………………...5 |  |
| 507 | How did you rate the advice that heath providers were providing on danger sign of the new born and the mother? | Strongly disagree…………………...1  Disagree………………………….....2  Neutral ……………………………..3  Agree……………………………….4  Strongly disagree…………………...5 |  |
| 508 | Did you understand what health care providers were told you? | Strongly disagree…………………...1  Disagree………………………….....2  Neutral ……………………………..3  Agree……………………………….4  Strongly disagree…………………...5 |  |
| 509 | Did you easily get the iron folate that was prescribed by health care providers? | Strongly disagree…………………...1  Disagree………………………….....2  Neutral ……………………………..3  Agree……………………………….4  Strongly disagree…………………...5 |  |
| 510 | Do you think the presence of traditional birth attendants during facility delivery is useful? | Strongly disagree…………………...1  Disagree………………………….....2  Neutral ……………………………..3  Agree……………………………….4  Strongly disagree…………………...5 |  |
| 511 | Do delivery services are provided free of charge? | Strongly disagree…………………...1  Disagree………………………….....2  Neutral ……………………………..3  Agree……………………………….4  Strongly disagree…………………...5 |  |
| 512 | Do post-natal services are provided free of charge? | Strongly disagree…………………...1  Disagree………………………….....2  Neutral ……………………………..3  Agree……………………………….4  Strongly disagree…………………...5 |  |
| 513 | Did your privacy was kept while you were deliver? | Strongly disagree…………………...1  Disagree………………………….....2  Neutral ……………………………..3  Agree……………………………….4  Strongly disagree…………………...5 |  |
| 514 | Did your privacy was kept while you got post-natal care services? | Strongly disagree…………………...1  Disagree………………………….....2  Neutral ……………………………..3  Agree……………………………….4  Strongly disagree…………………...5 |  |
| 515 | Was the general health service you got good? | Strongly disagree…………………...1  Disagree………………………….....2  Neutral ……………………………..3  Agree……………………………….4  Strongly disagree…………………...5 |  |

| **Social Support (SS)**  There are times in our lives when we need help, encouragement, and advice from people we know. This help can be given by a spouse, family members, friends, neighbors, or other members of your community. I would like to ask you questions about the different kinds of help you receive. | | | |
| --- | --- | --- | --- |
| **S.no** | **Question** | **Response Category** | **Skip** |
| 601 | Do you get visits from your friends, neighbors, and relatives (who do not live with you)? | Yes………………………...1  No…………………………0 |  |
| 602 | Do you get useful advice (from husband, family members, friends, neighbors, other members of your community) about important things in your life? | Yes………………………...1  No…………………………0 |  |
| 603 | Do you get to talk with someone (like your husband, family members, friends, neighbors, or other members of your community) you trust about your personal and family problems? | Yes………………………...1  No…………………………0 |  |
| 604 | Do you have people who care about what happens to you or would you like to have people who care about you? | Yes………………………...1  No…………………………0 |  |
| 605 | Do you feel loved by your family? | Yes………………………...1  No…………………………0 |  |
| 606 | Do your husband and family tell or show you that they are thankful for the things you do for your family? | Yes………………………...1  No…………………………0 |  |
| 607 | Do you get help with your household chores or Would you like to get help with chores? | Yes………………………...1  No…………………………0 |  |
| 608 | Do you get help with money in an emergency or would you like to have someone to help you? | Yes………………………...1  No…………………………0 |  |
| 609 | Does anyone help you or would you like someone to help when you need transportation? | Yes………………………...1  No…………………………0 |  |
| 610 | Do you get care or would like to be cared for when you are sick? | Yes………………………...1  No…………………………0 |  |
| 611 | Have you ever attended any group (i.e. 1-to 5 network, group women's groups; youth clubs, sports groups, trade unions, business or professional groups; self-help groups; and credit or savings groups? | Yes………………………...1  No…………………………0 |  |
| 612 | Are you a member in any religious, caste, or festival organization? | Yes………………………...1  No…………………………0 |  |
| 613 | Have you or anyone in the household attended a public meeting called by the village committee/women conference in the last year?” | Yes………………………...1  No…………………………0 |  |
| 614 | In this village/neighborhood, do people generally get along with each? | A conflict area …………………….1  Not a conflict area…………………2 | **End the interview.** |
