# Supplementary material for: Effect of checklist based box system interventions on improving institutional delivery among reproductive age women in Northwest Ethiopia: generalized structural equation modeling
Source: Arch Public Health. 2022 Jan 4;80:5. doi: 10.1186/s13690-021-00774-2 (PMC8725524; doi:10.1186/s13690-021-00774-2)
Supplement: Supplementary file 2 — Additional file 2. [file 13690_2021_774_MOESM2_ESM.docx]

| Structural Model | Unstandardized Path Coefficient | 95% CI |
| --- | --- | --- |
| Outcome: Institutional delivery |  |  |
| Checklist based box system intervention |  |  |
| Control (Ref) | -- | -- |
| Intervention | 1.20* | 0.86 – 1.54 |
| ANC four visit |  |  |
| No (Ref) | -- | -- |
| Yes | 0.33* | 0.02 – 0.65 |
| Danger Signs | -0.19 | - 0.47 -0.08 |
| BPCRp | -0.04 | - 0.25 - 0.18 |
| Social Support | 0.24* | 0.14 – 0.34 |
| Family Support | 0.07 | -0.07 – 0.22 |
| Influence |  |  |
| No (Ref) | -- | -- |
| Yes | -1.37* | -1.89 - -0.85 |
| Age | -3.38* | -4.31 - -2.44 |
| Place of Residence |  |  |
| Rural (Ref) | -- | -- |
| Urban | 0.28 | -0.22 – 0.77 |
| Indirect path: Antenatal care 4 attendance |  |  |
| Control (Ref) | -- | -- |
| Intervention | 1.73* | 1.43 – 2.03 |
| Month of ANC Initiation |  |  |
| >16 weeks of gestation (Ref) | -- | -- |
| ≤ 16 weeks of gestation | 0.68* | 0.36 – 0.99 |
| Social support | 0.05 | - 0.03 – 0.13 |
| Indirect path: BPCRp |  |  |
| Control (Ref) | -- | -- |
| Intervention | -0.18 | - 0.35 - 0.004 |
| Indirect path: Danger signs |  |  |
| Control (Ref) | -- | -- |
| Intervention | -1.92 | - 2.19 - 1.65 |
| Indirect Path: Month of ANC Initiation |  |  |
| Social Support | 0.45* | 0.37 – 0.53 |
| Indirect path: Danger signs |  |  |
| Social Support | 0.11* | 0.09 – 0.13 |

**Unstandardized path coefficients from the generalized structural equation modeling**
